# Multimethod geospatial modeling of life expectancy determinants in China

**DOI:** 10.3389/fpubh.2026.1810649

**Published:** 2026-05-07

**Authors:** Ke Hu, Xingjin Yang, Shuiping Ou, Chaojie Li, Xing Zhang, Di Xiao, Mingyang Yu

**Affiliations:** 1 Xiamen Haicang Hospital, Xiamen, Fujian, China; 2 QianDongNanZhou Center for Disease Control and Prevention, QianDongNanZhou, Guizhou, China; 3 Honwing Pharma (Guizhou) Company Limited, QianDongNanZhou, Guizhou, China; 4 Xingtai Center for Disease Control and Prevention, Xingtai, Hebei, China; 5 Nanjing Lishui Dongping Street Health Center, Nanjing, Jiangsu, China; 6 Community Health Service Center of Jiuxian Tongliang District, Chongqing, China; 7 Fuwai Central China Cardiovascular Hospital, Zhengzhou, Henan, China

**Keywords:** Geodetector, influencing factors, life expectancy, multiscale geographically weighted regression, spatial differentiation characteristics, spatial regression models

## Abstract

**Introduction:**

Although China has achieved sustained growth in life expectancy, significant spatial disparities persist across regions, necessitating investigation into their underlying determinants.

**Methods:**

This study systematically examines provincial-level life expectancy patterns in 2020 using multiple analytical approaches, including multiple linear regression (MLR), spatial lag model (SLM), geographically weighted regression (GWR), multiscale geographically weighted regression (MGWR), and Geodetector. The analysis incorporates comprehensive datasets covering socioeconomic indicators, education levels, healthcare resources, and environmental factors.

**Results:**

The findings reveal a pronounced east–west gradient, with coastal provinces exhibiting significantly higher life expectancy than inland regions. Spatial analysis identified distinct clustering patterns (Global Moran’s I = 0.5339, *p* < 0.001), with high-high clusters in eastern areas and low-low clusters in western regions. MGWR demonstrated superior performance by accounting for spatial variations in factor influences. Education level emerged as a fundamental determinant (PD = 0.850, *p* = 0.012), showing particularly strong effects in western China. Economic development also showed substantial explanatory power (PD = 0.661, *p* = 0.030), with greater influence in western provinces. Household consumption expenditure per capita (PD = 0.586, *p* = 0.027) and urbanization rate (PD = 0.633, *p* = 0.036) were additionally identified as significant factors. Household size showed a negative association exclusively in certain western regions. Notably, healthcare resource allocation demonstrated no significant effect (PD = 0.344, *p* = 1.000), while PM_2.5_ also lacked statistical significance (PD = 0.344, *p* = 1.000). Furthermore, Geodetector interaction analysis revealed that economic development and education level exhibited particularly strong synergistic effects (interaction PD = 0.944).

**Conclusion:**

The findings provide a scientific basis for formulating region-specific health policies and offer valuable insights for advancing the Healthy China initiative.

## 1 Introduction

As the world’s most populous country, China exhibits significant spatial disparities in life expectancy. While the national average life expectancy continues to rise, pronounced regional variations persist at the provincial level, demonstrating a distinctly uneven distribution pattern (1). For instance, economically developed coastal regions in eastern China generally report higher life expectancy than less developed western areas (2). Recent studies indicate a slight narrowing of these spatial disparities (3), yet the gap between the highest and lowest values remains as wide as 10 years. This enduring spatial heterogeneity underscores the necessity of investigating its underlying mechanisms from a geographical perspective, thereby providing scientific basis for formulating region-specific health policies (1).

The spatial heterogeneity of life expectancy results from the combined effects of multidimensional factors including socioeconomic conditions, environmental factors, and healthcare resources (4). As a core driving force, economic development level directly influenced health outcomes by affecting residents’ income (5), nutritional status (6), and accessibility to medical resources (7). Consumption level has also been demonstrated to impact life expectancy (8). The transformation of family structure (e.g., shrinking household size) indirectly affected health behaviors by altering family support systems (9). The uneven distribution of medical resources further exacerbated regional health disparities, serving as a key determinant of life expectancy (10). Additionally, environmental factors (e.g., air quality) exerted cumulative health effects through long-term exposure mechanisms (11–13), while urbanization shaped regional health patterns by modifying population density, infrastructure allocation, and lifestyle (14, 15). These factors not only functioned independently but could also collectively drive the spatial differentiation of life expectancy through complex interaction effects (4).

To quantify the effects of influencing factors and capture spatial dependence, this study employs multi-scale regression approaches. While traditional multiple linear regression (MLR) can identify global relationships among variables, it fails to address spatial autocorrelation issues (16). Global spatial regression models, including spatial error models (SEM) and spatial lag models (SLM), incorporate spatial dependence terms to effectively correct residual autocorrelation, thereby enhancing model robustness (17–19).

Geographically weighted regression (GWR) further overcomes the “spatial stationarity” assumption of conventional models by allowing regression coefficients to vary geographically, thereby revealing the spatial non-stationarity of influencing factors (20). Multiscale geographically weighted regression (MGWR) improves upon this by optimizing bandwidth selection for different variables, enabling more precise characterization of varying operational scales among factors (21). The Geodetector method specializes in analyzing both the independent explanatory power of individual factors and their interaction effects, proving particularly effective in identifying stratified heterogeneity of influencing factors (22).

However, the Healthy China initiative explicitly calls for “equal access to basic public health services” and “region-specific health strategies” to address persistent health inequalities across provinces. To directly advance this policy goal, it is insufficient to merely identify national-level determinants; rather, spatially explicit evidence is required to inform targeted interventions tailored to distinct regional contexts.

While previous studies have primarily relied on single analytical approaches—such as global regression models or geographically weighted regression alone—to examine the geographically heterogeneous distribution of life expectancy and its determinants, these isolated methods cannot simultaneously address the scale-dependent nature of different factors or detect the interactive effects among multiple determinants. To overcome these limitations, this study advances the existing methodological framework by integrating multiple spatial analytical techniques. Specifically, our research employs a comprehensive multi-tiered analytical framework ranging from global associations (MLR/SEM/SLM) to local variations (GWR/MGWR), and further to factor decomposition (Geodetector). The theoretical innovation lies in the coupled application of Multiscale Geographically Weighted Regression (MGWR)—which captures variable-specific spatial scales—and Geodetector—which quantifies factor interactions and stratified heterogeneity. This integrated approach not only identifies where each factor exerts its influence across different spatial scales but also reveals how factors work synergistically, thereby providing a more complete understanding of the spatial mechanisms underlying health inequalities.

## 2 Methods

### 2.1 Data sources

Building upon the theoretical framework established in the background section and considering data availability, this study selected seven key indicators for 2020 spanning multiple dimensions including economic development, family Structure, environment condition, education level, healthcare resources, consumption level, and urban–rural structure (see Table 1 for details). The analysis adopted provincial-level administrative divisions as study units, incorporating data from 31 provinces, autonomous regions, and municipalities directly under the central government in mainland China.

**Table 1:** Key factors selected for analysis.

Categories	Factors
Economic development	GDP per capita
Family structure	Average household size
Education level	Years of education
Healthcare resources	Number of hospital beds per 1,000 population
Environment condition	PM_2.5_
Consumption level	Household consumption expenditure per capita
Urban–rural structure	Urbanization rate

Life expectancy data were extracted from the China Health Statistical Yearbook (the most recent available data, as of 2020), while PM_2.5_ concentration values were obtained from provincial environmental status bulletins. All other explanatory variables were sourced from the China Statistical Yearbook. All variables refer to the year 2020.

### 2.2 Spatial autocorrelation analysis

Spatial autocorrelation analysis was conducted to evaluate the distribution characteristics of spatial data using both global and local Moran’s indices. A first-order queen contiguity spatial weight matrix was constructed based on provincial-level administrative boundaries, where neighboring provinces sharing at least one common vertex were assigned a weight of 1 and 0 otherwise. The matrix was row-standardized. This weight matrix was used for global Moran’s I and Local Moran’s Index (LISA).

#### 2.2.1 Global Moran’s I

The Global Moran’s I index was employed to assess the overall spatial clustering pattern across the study area. The mathematical expression is given by Equation 1 (23):


$$I=\frac{n\sum_{i=1}^{n}\sum_{j=1}^{n}W_{\mathrm{ij}}(x_{i}-\bar{x})(x_{j}-\bar{x})}{\sum_{i=1}^{n}\sum_{j=1}^{n}W_{\mathrm{ij}}\sum_{i=1}^{n}{\left(x_{i}-\bar{x}\right)}^{2}}$$ (1)

The Global Moran’s I index ranges from −1 to 1, where *n* is the sample size, *W*_ij_ represents the elements of the spatial weight matrix, *x*_i_ and *x*_j_ denote the observed values, and $\bar{x}$ is the mean value. A positive correlation (0 < *I* ≤ 1) indicates spatial clustering of similar values, while a negative correlation (−1 ≤ *I* < 0) suggests adjacent distribution of high and low values. When *I* = 0, the spatial pattern shows random distribution with no significant correlation. The statistical significance of these results must be verified through Z-test (24).

#### 2.2.2 Local Moran’s Index (LISA)

LISA were employed to identify local spatial heterogeneity patterns. The calculation formula is expressed as Equation 2 (1):


$$I_{i}=\frac{n(x_{i}-\bar{x})\sum_{j=1}^{n}w_{\mathrm{ij}}(n_{j}-\bar{n})}{\sum_{j=1}^{n}{\left(x_{j}-\bar{x}\right)}^{2}}$$ (2)

LISA adopts the same variable definition framework as the Global Moran’s I index to identify four characteristic spatial patterns: HH-type (high-value clusters), LL-type (low-value clusters), HL-type (high values surrounded by low values), and LH-type (low values surrounded by high values). The combination of significance testing (*p* < 0.05) and spatial visualization techniques effectively reveals distinct spatial heterogeneity characteristics (25).

### 2.3 Multiple linear regression (MLR)

MLR model was employed to analyze the collective influence of multiple independent variables on a single dependent variable, with the basic model formulated as Equation 3 (26):


$$Y=\beta_{0}+\beta_{1}X_{1}+\beta_{2}X_{2}+\ldots +\beta_{p}X_{p}+\varepsilon$$ (3)

Where *Y* represents the dependent variable, *X*_1_ to *X*_p_ denote the independent variables, *β*_0_ is the intercept term, *β*_1_ to *β*_p_ are regression coefficients, and *ε* signifies the normally distributed random error term with zero mean. Model performance was comprehensively assessed through the coefficient of determination (*R*^2^) measuring explanatory power, the Akaike Information Criterion (AIC) evaluating the trade-off between model complexity and goodness-of-fit, and variance inflation factors (VIF) testing for multicollinearity, where VIF values exceeding 5 indicate significant multicollinearity that may compromise model reliability (27).

### 2.4 Global spatial regression models

The same first-order queen contiguity spatial weight matrix described in the spatial autocorrelation analysis was used for the global spatial regression models.

#### 2.4.1 Spatial error model (SEM)

The SEM was employed to address spatial autocorrelation in regression residuals, with its basic formulation expressed as Equations 4, 5:


$$Y=X_{\beta }+u$$ (4)

$$u=\lambda W_{u}+\varepsilon$$ (5)

Where *λ* represents the spatial error coefficient and *W*_u_ denotes the spatially lagged error term. This model specification effectively resolves estimation bias caused by spatial autocorrelation in conventional regression analysis by explicitly incorporating spatial dependence structure in the error terms (18).

#### 2.4.2 Spatial lag model (SLM)

SLM captures spatial dependence effects by incorporating a spatially lagged term of the dependent variable, effectively characterizing the interaction mechanisms between adjacent spatial units. The model specification takes the form Equation 6 (28):


$$Y=\rho W_{Y}+X_{\beta }+\varepsilon$$ (6)

Where *ρ* denotes the spatial autoregressive coefficient that quantifies the strength of spatial dependence, *W* represents the row-standardized spatial weight matrix defining neighborhood relationships, and *W*_Y_ constitutes the spatially lagged dependent variable. The SLM framework explicitly accounts for spillover effects where the dependent variable in a given location is influenced by values of this variable in neighboring locations, while simultaneously controlling for the effects of local explanatory variables through the *β* coefficient vector.

### 2.5 Local spatial regression models

#### 2.5.1 Geographically weighted regression (GWR)

GWR model addresses spatial non-stationarity through local regression analysis, differing from traditional global regression by permitting spatially varying regression coefficients (1). The basic GWR model formulation is Equation 7:


$$Y_{i}=\beta_{0}(u_{i},v_{i})+\sum_{k=1}^{m}\beta_{k}(u_{i},v_{i})x_{\mathrm{ik}}+\varepsilon_{i}$$ (7)

Where (*u*_i_,*v*_i_) represents the spatial coordinates of observation *i*, and *β*_k_(*u*_i_,*v*_i_) denotes the location-specific regression coefficients. The model calculates spatial weights using a distance decay function and estimates parameters through weighted least squares. In this study, a fixed Gaussian kernel was employed for GWR, consistent with the specification used in MGWR to ensure methodological comparability.

#### 2.5.2 Multiscale geographically weighted regression (MGWR)

MGWR extends GWR by allowing different explanatory variables to operate at distinct spatial scales. Its formulation is Equation 8:


$$Y_{i}=\beta_{0}\;(u_{i},v_{i},bw_{0}\;)+\sum_{k=1}^{m}\beta_{k}\;(u_{i},v_{i},b_{\mathrm{wk}}\;)x_{\mathrm{ik}}+\varepsilon_{i}\;$$ (8)

Where *bw*_k_ represents the bandwidth parameter for the *k*_th_ variable. MGWR determines optimal bandwidths for each variable via an iterative back-fitting algorithm, providing more accurate characterization of complex spatial dependence patterns (21). Compared to GWR, MGWR offers superior performance by eliminating single-bandwidth-induced estimation bias, identifying variable-specific spatial scales, and enhancing interpretation accuracy. This study implemented a fixed Gaussian kernel function with bandwidth selection based on the AIC_c_ criterion. Given the relatively uniform spatial distribution of provincial-level administrative units in terms of geographic extent and density, a fixed Gaussian kernel is methodologically appropriate as it assumes a constant spatial scale of influence across the study area. Under such conditions, kernel function choice typically has minimal impact on coefficient estimates, and the fixed kernel has been widely adopted in similar provincial-level health geography studies.

### 2.6 Geodetector

Geodetector is a statistical analysis method based on the theory of spatial stratified heterogeneity (29–31). Through four interrelated functional modules, it systematically reveals the spatial differentiation characteristics of geographical elements and their driving mechanisms:

The factor detector quantitatively measures the explanatory power of various independent variables on geographical phenomena using PD-statistics (Ranging from 0 to 1). A higher PD-value indicates stronger explanatory capability, with PD = 1 representing complete explanation of the spatial variation. The mathematical formulation of the model is presented in Equation 9 (32):


$$\mathrm{PD}=1-\frac{\sum_{h=1}^{L}N_{h}\sigma_{h}^{2}}{N\sigma^{2}}$$ (9)

where *N*_h_ represents the sample size of the *h*-th stratum, *σ*_h_^2^ denotes the variance within the *h*-th stratum, *N* stands for the total sample size, and *σ*^2^ indicates the total variance across all strata.

The interaction detector identifies and quantifies how the combined effect of two factors on an outcome variable differs from the sum of their individual effects, revealing synergistic or antagonistic interactions. The interaction types can be found in Table 2 (4).

**Table 2:** Interaction types between variables.

Description	Interaction
$\mathrm{PD}(X_{1}\cap X_{2})<\min(\mathrm{PD}(X_{1}),\mathrm{PD}(X_{2}))$	Weaken, nonlinear
$\begin{array}{l} \min(\mathrm{PD}(X_{1}),\mathrm{PD}(X_{2}))\\ <\mathrm{PD}(X_{1}\cap X_{2})\\ <\max(\mathrm{PD}(X_{1})),\mathrm{PD}(X_{2})) \end{array}$	Weaken, univariate
$\mathrm{PD}(X_{1}\cap X_{2})>\max(\mathrm{PD}(X_{1}),\mathrm{PD}(X_{2}))$	Enhanced, bivariate
$\mathrm{PD}(X_{1}\cap X_{2})=\mathrm{PD}(X_{1})+\mathrm{PD}(X_{2})$	Independent
$\mathrm{PD}(X_{1}\cap X_{2})>\mathrm{PD}(X_{1})+\mathrm{PD}(X_{2})$	Enhance, nonlinear

The risk detector employs *T*-tests to examine spatial heterogeneity characteristics and identify statistically significant high-risk and low-risk zones (29).

The ecological detector applies F-tests to compare spatial distribution differences among influencing factors and determine their statistical significance levels.

This methodology offers three notable advantages: it requires no linearity assumptions and can effectively capture complex nonlinear relationships; it inherently avoids multicollinearity issues common in traditional regression models; and it produces results with clear geographical significance for spatial decision-making. Currently, the method has been widely applied in land use/cover change studies, environmental health risk assessments, and regional economic disparity analyses.

### 2.7 Independent variable discretization methods

As Geodetector requires categorical input variables, discretization of continuous variables becomes a crucial preprocessing step. This study comprehensively compares five classical spatial discretization methods:

Natural breaks: Determining optimal segmentation points based on inherent data distribution characteristics to maximize inter-class differences.
Quantile classification: Ensuring balanced sample sizes across categories, particularly suitable for right- or left-skewed distribution data.
Equal interval classification: Dividing the value range into equal intervals, applicable to variables with uniform distribution characteristics.
Geometric interval classification: Using geometric progression for interval division, effectively handling exponentially distributed data.
Standard deviation classification: Defining boundaries by mean±n standard deviations, specifically designed for normally distributed data.

The discretization process strictly follows the “explanatory power maximization” principle, systematically comparing the factor detection performance of different classification methods (3–8 classes) to ultimately determine the optimal discretization scheme (33).

### 2.8 Software

The study employed ArcGIS 10.2 software for independent variable discretization, spatial correlation analysis, collinearity diagnostics, and cartographic visualization. GeoDa 1.22 software was utilized to construct MLR, SEM and SLM, while MGWR 2.2 software was applied for GWR and MGWR analyses. Geodetector analysis was conducted using its official Excel version (available at: http://geodetector.cn/Download.html). All base map data were sourced from the National Platform for Common Geospatial Information Services (approval number: GS(2024)0650). Statistical analyses employed two-tailed tests with a significance level set at *p* < 0.05.

## 3 Results

### 3.1 Results of spatial autocorrelation analysis

**Figure 1:**
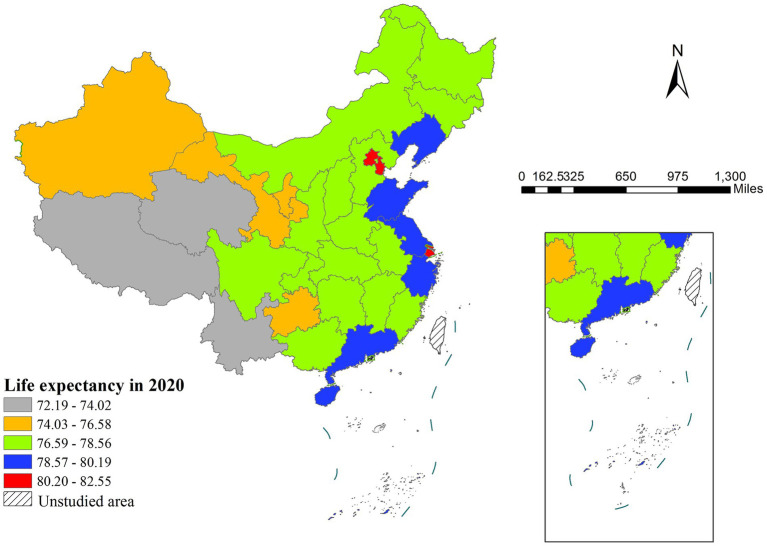
The spatial distribution of life expectancy in China (2020).

The results revealed significant spatial disparities in life expectancy across China in 2020. Eastern regions consistently showed higher longevity, led by Shanghai (82.49 years), while western areas lagged behind, with Tibet recording the lowest value (72.19 years)—a 10.3-year gap that reflected regional development inequalities (Figure 1).

**Figure 2:**
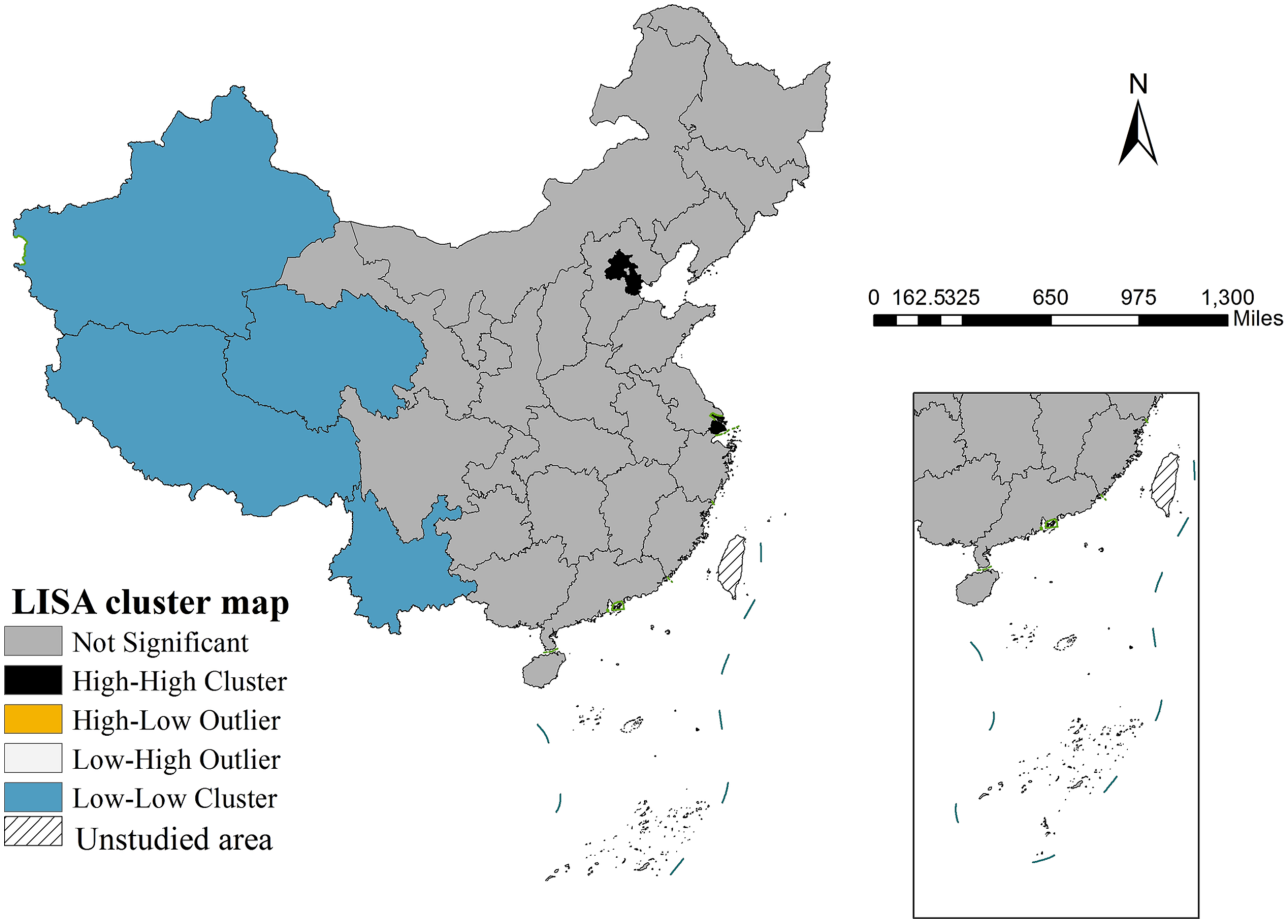
LISA cluster map of life expectancy (2020).

Spatial autocorrelation analysis demonstrated strong clustering patterns, as evidenced by a Moran’s I coefficient of 0.5339 (*p* < 0.001), indicating pronounced positive spatial dependence in life expectancy distribution. Local spatial autocorrelation analysis identified two distinct clustering patterns: high-high clusters concentrated in the Beijing-Tianjin-Shanghai metropolitan region and low-low clusters predominantly located in the western provinces of Tibet, Xinjiang, Qinghai and Yunnan (Figure 2).

### 3.2 Results of global regression model

The MLR results initially revealed significant multicollinearity between household consumption expenditure and urbanization rate with other variables (both with VIF values >14). After excluding these two factors, all remaining variables showed VIF values below 4. The preliminary multiple linear regression analysis (Table 3) indicated that only years of education demonstrated statistically significant effects on life expectancy. However, the Moran’s I test of regression residuals revealed significant spatial autocorrelation (*p* = 0.00225), suggesting that conventional MLR models might fail to fully capture the complex spatial relationships affecting life expectancy. The Lagrange Multiplier (LM) diagnostic tests revealed significance for both the LM lag and robust LM lag statistics (*p* < 0.05), while the LM error and robust LM error statistics were non significant (*p* > 0.05), suggesting that a spatial lag model (SLM) is preferred over a spatial error model (SEM) for subsequent analysis. This finding implies that life expectancy in neighboring regions exhibits spatial dependence that should be explicitly modeled.

**Table 3:** Results of the SLM and MLR.

Factors	SLM		MLR	
	Coefficient	*p*-value	Coefficient	*p*-value
Intercept	0.03486	0.57484	−0.00000	1.00000
GDP per capita	0.08515	0.40998	0.14877	0.28728
Average household size	−0.08363	0.41475	−0.14284	0.28108
Years of education	0.58778	0.00000	0.656102	0.00056
Number of hospital beds per 1,000 population	−0.06009	0.48930	−0.145811	0.17990
PM_2.5_	−0.00647	0.93333	0.027195	0.79013

SLM results revealed a significant spatial dependence in life expectancy patterns, with the spatial lag coefficient (*ρ*) being 0.054 (*p* = 0.00064), indicating notable spatial autocorrelation among neighboring regions. As shown in Table 3, only years of education demonstrated statistically significant effects on life expectancy in the SLM framework. Compared to the MLR, the variable of education level exhibits a smaller *p*-value in SLM, suggesting enhanced statistical significance. The SLM demonstrates superior model performance with higher *R*^2^ and log-likelihood values, along with lower AIC, indicating better goodness-of-fit. Importantly, the Moran’s I test of residuals becomes statistically insignificant after accounting for spatial effects, confirming that the SLM has effectively captured the underlying spatial structure without omitting critical spatial autocorrelation information. These findings collectively validate the necessity of incorporating spatial effects when analyzing geographical variations in life expectancy. Given the LM test results favoring SLM over SEM, the SLM was selected as the representative global spatial model for subsequent comparisons with local models.

### 3.3 Results of local spatial regression model

This study developed both GWR and MGWR models, with comparative analysis of model goodness-of-fit demonstrating that the MGWR model outperformed the GWR model in fitting performance (Table 4). Further comparisons revealed that both local spatial regression models showed significantly better fitting results than the SLM, with neither exhibiting spatial autocorrelation in their residuals. Consequently, this study selected the MGWR model to examine the differential impacts of various factors on life expectancy across provinces.

**Table 4:** Goodness-of-fit of the four models.

Goodness-of-fit	MGWR	GWR	SLM	MLR
Moran’s I	−0.09 (*p* = 0.572)	−0.06 (*p* = 0.778)	−0.040 (*p* = 0.95)	0.2185 (*p* = 0.0025)
*R* ^2^	0.951	0.909	0.878	0.827
AIC	20.868	34.547	37.126	44.601
Log likelihood	2.869	−6.870	−11.563	−16.301

**Figure 3:**
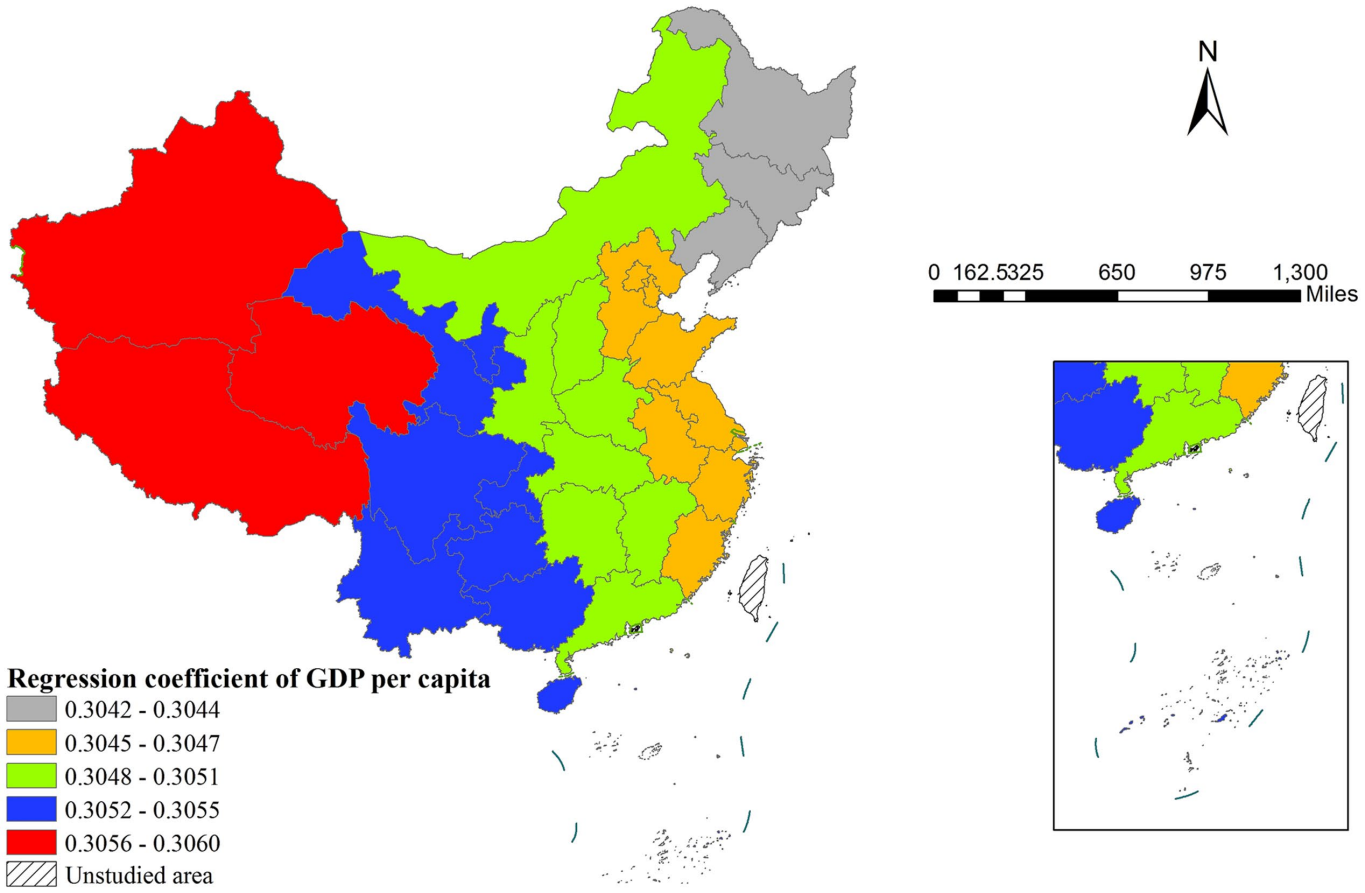
The spatial distribution of regression coefficient of GDP per capita.

**Figure 4:**
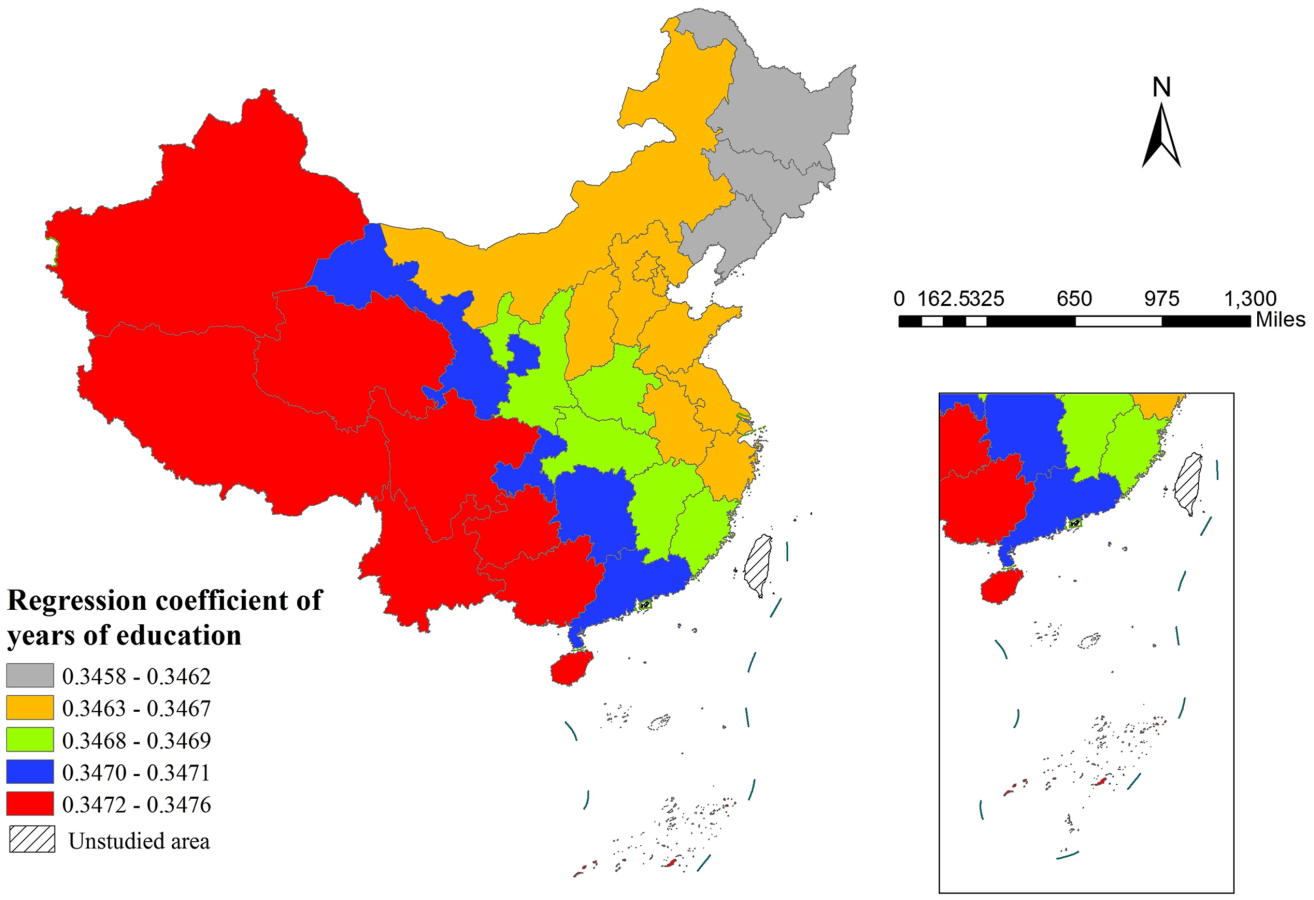
The spatial distribution of regression coefficient of years of education.

The MGWR model results indicated that four out of five variables influenced life expectancy, showing distinct spatial heterogeneity in their effects. GDP per capita demonstrated particularly notable regional variations in its impact on life expectancy. The regression coefficients for GDP per capita were not only statistically significant in western regions but also substantially larger than those in eastern areas (Figure 3), suggesting that economic development plays a more pronounced role in improving health outcomes in less developed western provinces. Similarly, years of education showed the strongest positive association in western China and the weakest effect in eastern regions (Figure 4).

Notably, while the analysis revealed clear east–west gradients in both life expectancy and its determinants, no statistically significant north–south disparities were detected. This finding does not necessarily imply the absence of north–south differences in reality, but rather suggests that such differences—if they exist—may be less pronounced than the dominant east–west gradient, or may operate at spatial scales not fully captured by provincial-level analysis. The strong spatial clustering patterns identified by LISA (high-high in eastern metropolitan regions, low-low in western provinces) further support the primacy of the east–west divide in shaping China’s health geography.

**Figure 5:**
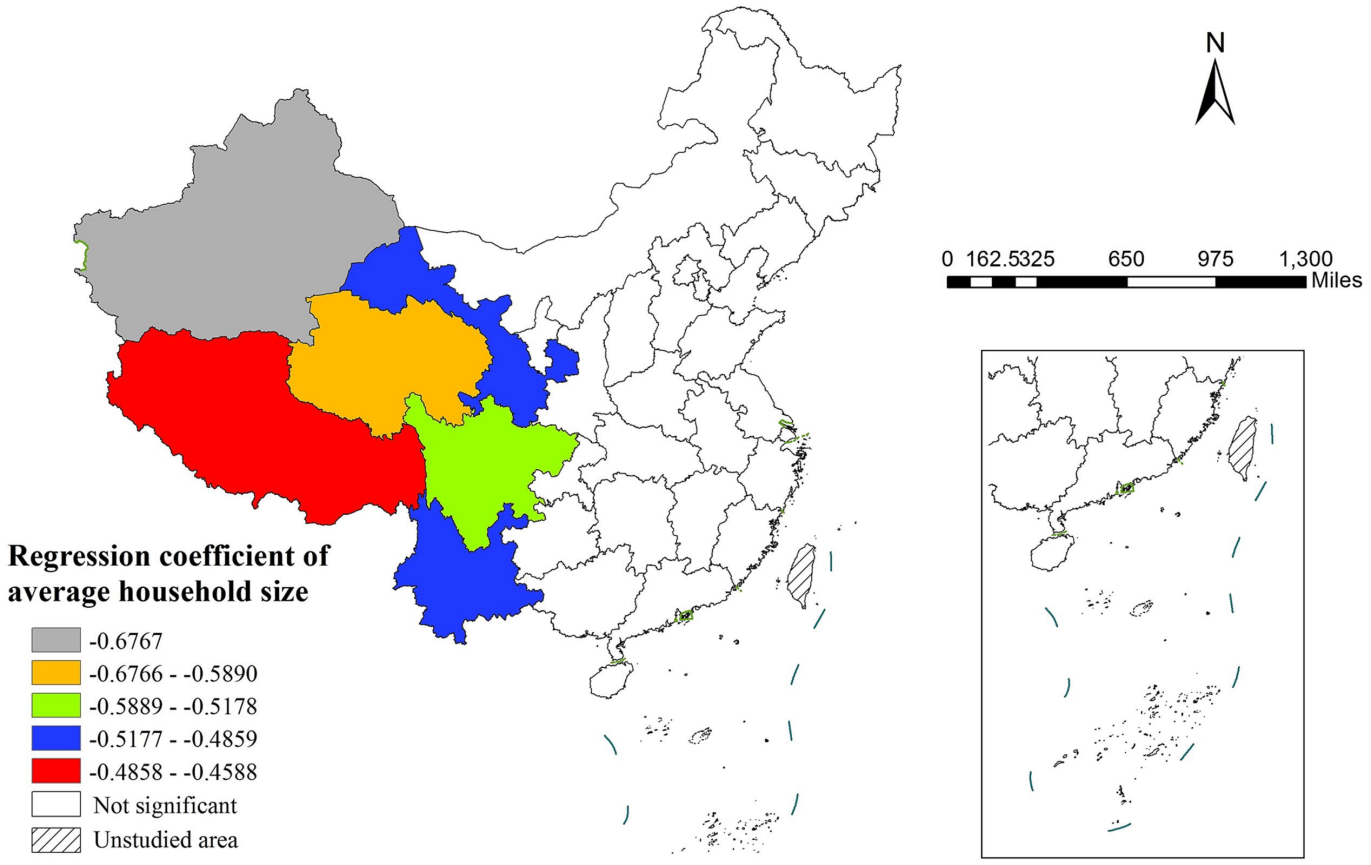
The spatial distribution of regression coefficient of average household size.

**Figure 6:**
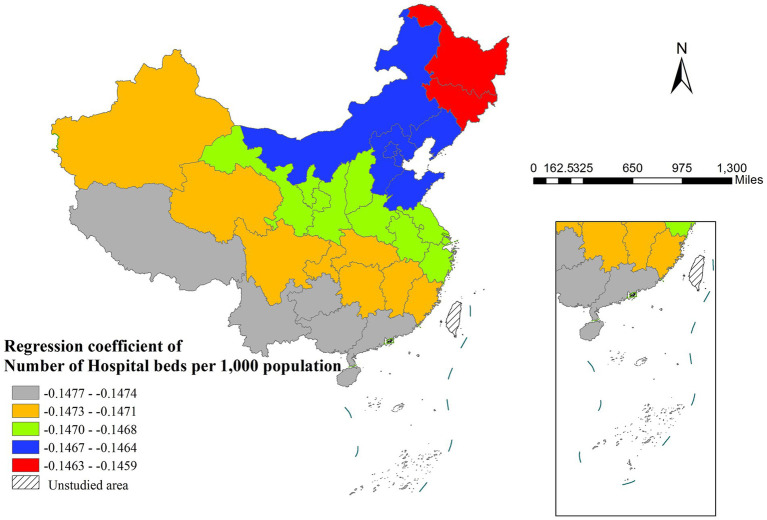
The spatial distribution of regression coefficient of number of hospital beds per 1,000 population.

Average household size exhibited spatial variability in its influence, displaying significant negative effects only in certain western areas while showing no statistically significant impact in central and eastern regions (Figure 5). Notably, healthcare resource availability (measured by hospital beds per 1,000 population) showed an unexpected negative correlation with life expectancy, particularly strong in southwestern China and relatively weaker in northeastern regions (Figure 6). Environmental factors represented by PM_2.5_ concentration failed to achieve statistical significance in any study region, suggesting their potential effects might be overshadowed by other variables.

**Figure 7:**
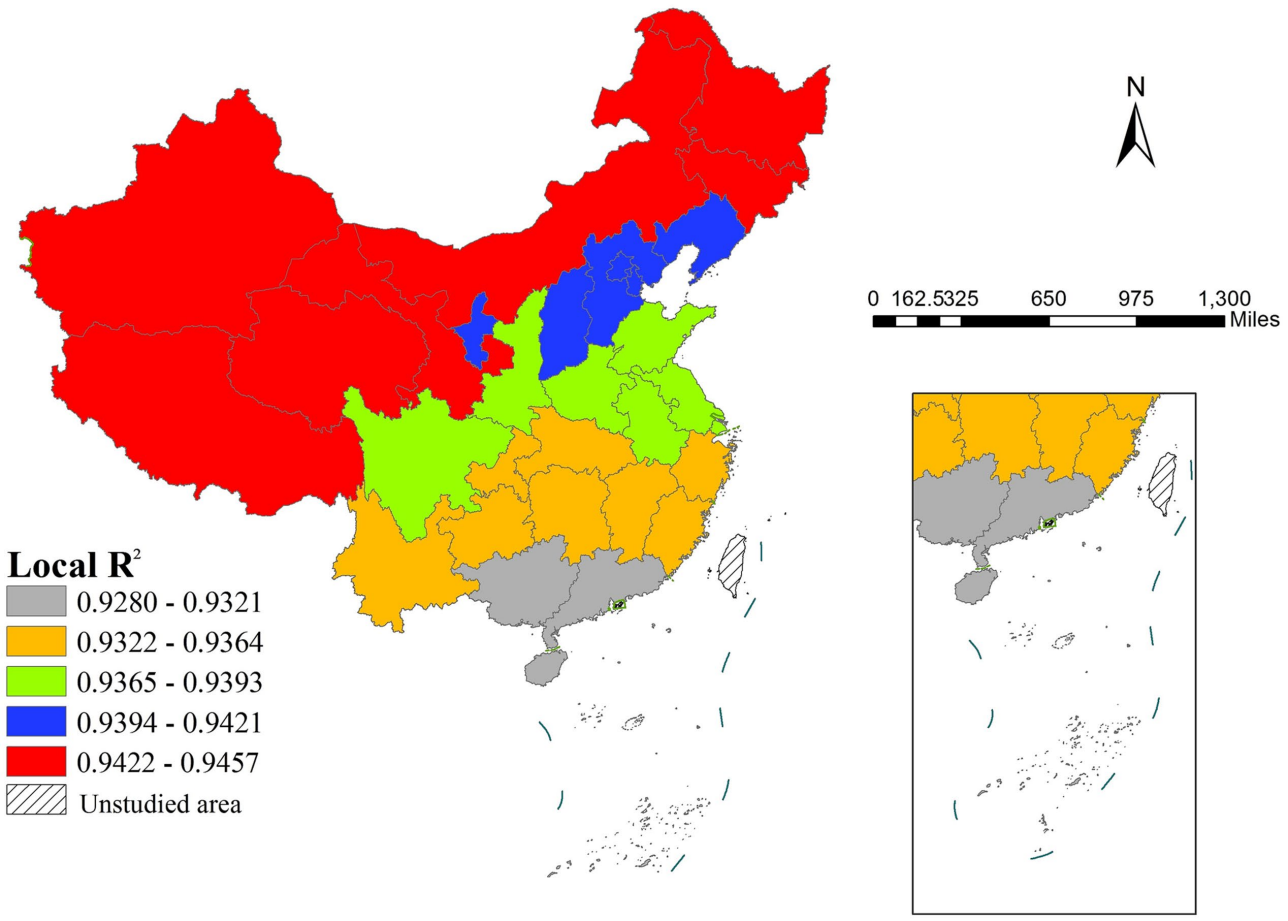
The spatial distribution of local *R*^2^.

Model evaluation revealed generally higher local *R*^2^ values in northern China (Figure 7), indicating stronger explanatory power of the selected variables for life expectancy variation in these areas. In contrast, the model showed relatively poorer fit in southern regions, suggesting the potential need to incorporate additional explanatory variables to enhance model performance in these areas. This north south variation in model fit does not detract from our east west focused findings, as the primary determinants driving the east west life expectancy gradient remain well captured by the selected variables.

### 3.4 Results of Geodetector

#### 3.4.1 Discretization of independent variables

The study employed five classification methods to discretize seven continuous independent variables, with the number of categories ranging from 3 to 8. Through systematic comparison using the factor detector, the optimal classification scheme was determined by selecting the approach yielding the maximum PD value for each variable (see Table 5 for details).

**Table 5:** Results of factor detection and variable classification.

Factors	PD	*p*	Classification method	Classification interval
GDP per capita	0.661	0.030	Quantile	6
Average household size	0.442	0.000	Quantile	3
Years of education	0.850	0.012	Quantile	8
Number of hospital beds per 1,000 population	0.344	1	Natural breaks	7
PM_2.5_	0.344	1	Quantile	8
Household consumption expenditure per capita	0.586	0.027	Quantile	4
Urbanization rate	0.633	0.036	Quantile	5

#### 3.4.2 Factor detector results

The results in Table 5 demonstrate that GDP per capita, household size, and years of education significantly influence life expectancy, along with household consumption expenditure per capita and urbanization rate which were previously excluded in other models due to multicollinearity concerns. Unlike regression models that suffer from multicollinearity among these variables, Geodetector is immune to such issues and therefore can validly include these factors even though they were excluded from the MLR and SLM due to multicollinearity concerns. Notably, in contrast to the MGWR model findings, the number of hospital beds per 1,000 population showed no significant effect on life expectancy in this analysis.

#### 3.4.3 Risk detector results

**Figure 8:**
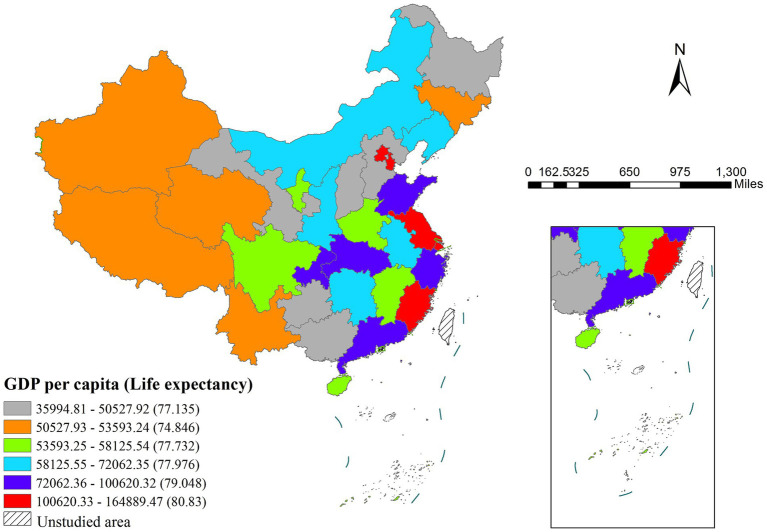
Regional disparities in GDP per capita and associated life expectancy.

The risk detector quantified average life expectancy across different strata of influencing factors and assessed the statistical significance of inter-strata differences. As illustrated in Figure 8, GDP per capita exhibited a clear association with life expectancy. The lowest life expectancy (77.135 years) occurred in the lowest GDP stratum (35,994.81–50,527.92 yuan), while the peak life expectancy (80.83 years) was observed in the highest GDP stratum (100620.33–164889.47yuan).

Table 6 presents the significance test results (Y/N) for life expectancy differences across GDP strata. When GDP per capita was divided into six gradients, statistical analysis revealed significant differences in life expectancy between the highest stratum (level 6) and the four lowest strata (levels 1–4).

**Table 6:** Significance of differences in mean life expectancy across GDP per capita Strata.

Strata	1	2	3	4	5	6
1						
2	N					
3	N	Y				
4	N	Y	N			
5	Y	Y	Y	Y		
6	Y	Y	Y	Y	N	

**Figure 9:**
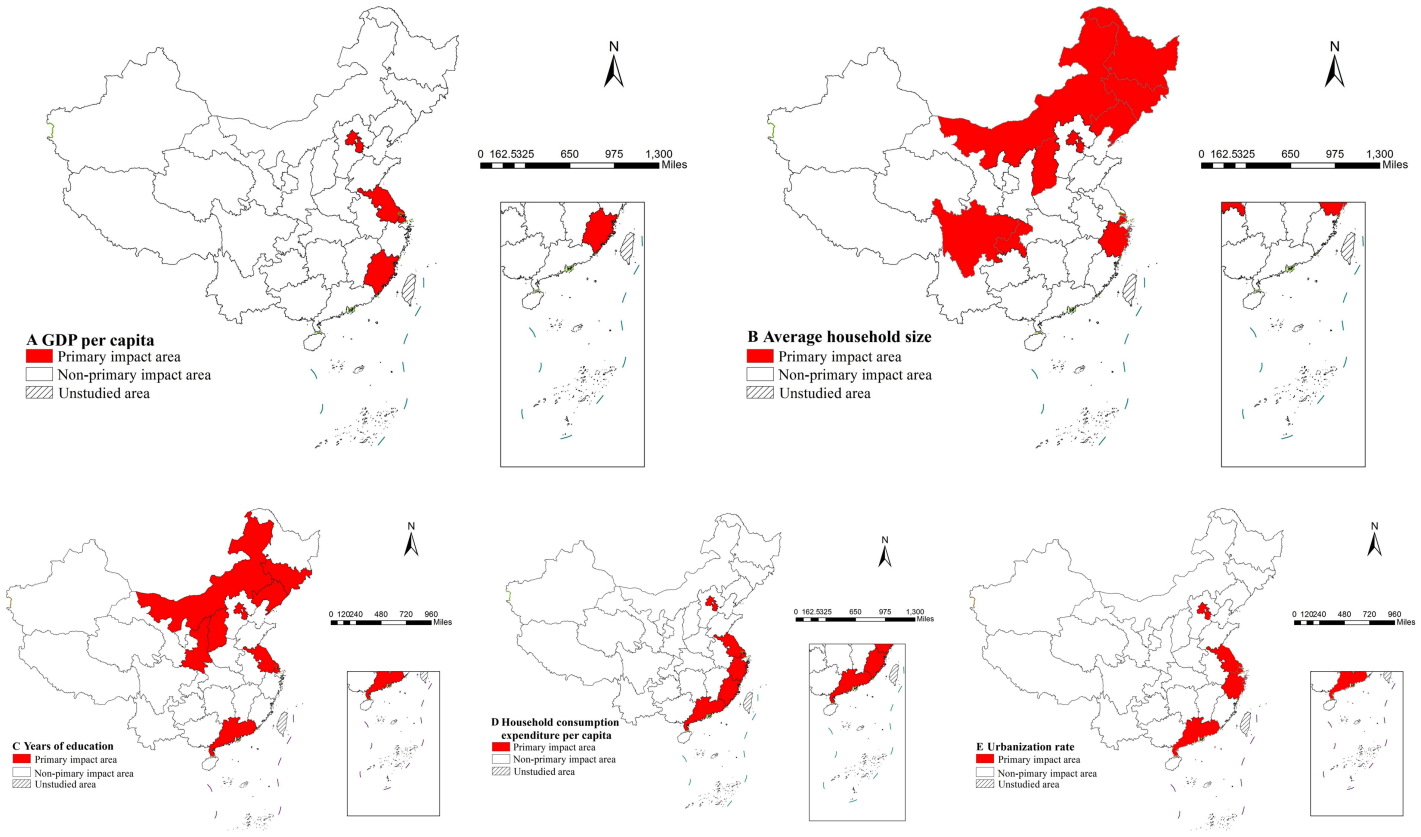
Distribution of main impact area of each factors.

The risk detector further enabled quantitative assessment of factor and life expectancy relationships. As shown in Table 7, each factor exhibited an optimal range corresponding to maximum life expectancy values (34). From an epidemiological perspective, these optimal ranges define the principal domains of influence for each factor (35). The spatial characteristics of these quantitative relationships were visually represented through geospatial mapping in Figure 9.

**Table 7:** Optimal range of factors and their corresponding maximum life expectancy.

Factors	Optimal interval	Maximum life expectancy (Years)
GDP per capita (RMB)	100620.33–164889.47	80.830
Average household size	2.22–2.52	79.426
Years of education	9.70–12.21	82.113
Household consumption expenditure per capita (RMB)	216178.15–42536.29	80.521
Urbanization rate (%)	72.15–89.3	80.860

#### 3.4.4 Ecological detector results

The ecological detector conducted pairwise comparative analysis of key factors affecting life expectancy. As shown in Table 8, the statistical analysis results indicated that the differences in PD values between years of education and all other factors were statistically significant (marked as “Y”), while most differences in PD values between other variables were not statistically significant. To further investigate the interaction effects among influencing factors and their synergistic mechanisms on life expectancy, subsequent in-depth analysis will be conducted using the interaction detector.

**Table 8:** Significance testing for pd value differences between factors.

Factors	GDP per capita	Average household size	Years of education	Household consumption expenditure per capita	Urbanization rate
GDP per capita					
Average household size	Y				
Years of education	Y	Y			
Household consumption expenditure per capita	N	N	Y		
Urbanization rate	N	N	Y	N	

#### 3.4.5 Interaction detector results

This study found significant synergistic effects among various influencing factors through interaction detector analysis. The results showed that the interactive PD values of all pairwise factor combinations were significantly higher than the independent effects of single factors, with the interaction effect between GDP per capita and years of education being the most significant (PD = 0.944) (Table 9). Notably, all pairwise variable combinations exhibited typical bifactor enhancement effects. This finding provides a new perspective for understanding the influencing factors of life expectancy and emphasizes the importance of comprehensive consideration of multiple dimensions and factors.

**Table 9:** Interaction effects between factors on life expectancy.

Factors	GDP per capita	Average household size	Years of education	Household consumption expenditure per capita	Urbanization rate
GDP per capita	0.661				
Average household size	0.894	0.442			
Years of education	0.944	0.902	0.850		
Household consumption expenditure per capita	0.747	0.754	0.936	0.586	
Urbanization rate	0.871	0.713	0.943	0.683	0.633

## 4 Discussion

This study adopts a multidimensional analytical framework integrating MLR, SLM, GWR, MGWR models, combined with Geodetector model, to examine the influencing factors of life expectancy.

Notably, the MGWR model effectively captures the spatial-scale heterogeneity of explanatory variables through its variable bandwidth parameters. Meanwhile, the Geodetector provides robust validation by quantifying the determinant power (PD-value) of each factor and revealing potential interactive effects among variables.

The synergistic application of these advanced spatial statistical techniques enables a more nuanced understanding of regional disparities in life expectancy. This integrated methodology not only overcomes the limitations of traditional global regression models but also provides policymakers with spatially explicit evidence for targeted health interventions.

The MGWR results reveal that the coefficients of GDP per capita and years of education on life expectancy are higher in western regions than in eastern regions, indicating that economic development and education level exert a more pronounced effect on life expectancy improvement in the west. In the economically advanced east, where GDP per capita is already high, its marginal health benefits may exhibit diminishing returns (36). In contrast, in the less-developed west, GDP growth leads to more significant enhancements in healthcare resources and public health (2). Similarly, education quality shows regional heterogeneity—its health benefits are more substantial in the west, whereas the east may be approaching the threshold of educational returns (37).

Average household size negatively impacts life expectancy, as larger families may lead to overcrowded living conditions, increasing stress for members of joint or extended households (38). This effect is observed only in certain western areas, with negligible influence in central and eastern regions. This disparity may stem from the heavier economic burden imposed by larger households in the west, whereas the more robust social security systems in central and eastern regions alleviate such pressures.

The negative correlation between the number of hospital beds and life expectancy may reflect potential inefficiencies in resource utilization and imbalances in healthcare allocation. An excessive number of beds could lead to the dispersion of medical resources (e.g., doctors and nurses), thereby reducing overall healthcare quality (39). This effect is more pronounced in the southwestern region, where the regression coefficient is higher, compared to the northeastern region, where it is lower. This disparity likely stems from uneven distribution of medical resources in the southwest, resulting in low bed utilization rates, whereas the relatively balanced healthcare system in the northeast ensures that bed supply aligns more closely with actual demand. Additionally, patient cross-regional mobility—where residents from southwestern areas seek treatment in more developed neighboring provinces—may further weaken the association between local healthcare resources and resident life expectancy. The Geodetector results further corroborate this finding, showing no significant impact of hospital bed numbers on life expectancy, which underscores the inefficiency in healthcare resource allocation.

Years of education consistently demonstrate a significant influence across MLR, SLM, and MGWR models. Furthermore, the factor and ecological analyses of Geodetector reveal that education exerts a substantially stronger effect on life expectancy than other variables. Compared to GDP growth, expanding educational coverage more directly reduces disparities in life expectancy (40, 41). Education not only directly influences individuals’ health behaviors and access to medical resources but also exerts a more profound and enduring impact on life expectancy than purely economic factors through multiple pathways, such as altering socioeconomic status, environmental exposures, and stress levels (41–44).

Despite economic imbalances between northern and southern China, our analysis did not detect statistically significant north–south disparities in life expectancy determinants. This may be attributed to three factors: First, the dominant east–west gradient—rooted in China’s coastal-first development policies—may overshadow potential north–south variations. Second, the provincial-level analytical scale may aggregate and smooth over intra-provincial north–south differences, particularly in large provinces that span both northern and southern climatic or cultural zones (e.g., Jiangsu, Anhui, Henan). Third, the local *R*^2^ values were notably higher in northern than southern regions, suggesting that our selected variables explain life expectancy variation more effectively in northern areas, while southern regions may require additional explanatory factors not captured in this study. Future research incorporating finer-scale data and region-specific variables could better reveal potential north–south disparities.

Interaction analysis based on the Geodetector reveals that synergistic effects among influencing factors significantly surpass the independent effects of single variables on regional health outcomes. Among various combinations, the interaction between economic development and education level exhibits the most pronounced synergistic effect. This may operate through a bidirectional reinforcement mechanism: economic growth provides the material foundation for optimizing medical resources and improving living conditions, while enhanced education fosters health-conscious behaviors by strengthening health literacy and disease prevention awareness. The two factors exhibit a clear positive feedback relationship, jointly forming a composite driving system for regional health development.

This study innovatively establishes a coupled analytical framework integrating MGWR and Geodetector models, overcoming the limitations of traditional spatial regression models in identifying variable spatial heterogeneity while resolving the challenges in detecting interaction effects among influencing factors. This multidimensional, multi-method integrated innovation not only expands the research paradigm of health geography but also provides a novel analytical tool for the precise implementation of public health policies.

Several limitations of this study should be acknowledged: First, regarding data, constrained by the availability of statistical records, the adopted analytical units (e.g., provincial-level scale) may obscure heterogeneity characteristics at finer spatial resolutions. While provincial-level analysis is currently the most feasible approach given that official life expectancy data from the 2020 Population Census are only published at this scale, we acknowledge that this may mask within-province variations, particularly in large provinces with uneven development. Future studies should examine city- or county-level patterns should such data become available. The life expectancy data are sourced from the authoritative 2020 China Population Census, the most comprehensive dataset available for provincial-level analysis. Although five-year intercensal surveys exist, their detailed life expectancy data have not been released. Thus, while we acknowledge this timeliness limitation, the 2020 census remains the most reliable source for this study, and future research should incorporate newer data when available. Additionally, certain potentially important variables (such as meteorological factors like temperature and humidity) were excluded from the model due to data accessibility challenges. These omitted climatic factors may partly explain the lower model fit observed in southern China. Second, the modest sample size (*n* = 31 provinces) may increase the risk of overfitting in local regression models such as MGWR. Nevertheless, the AICc based bandwidth selection procedure inherently penalizes model complexity, and the non significant residual Moran’s I suggests adequate model specification. Future studies with larger samples or higher resolution spatial units would help confirm the robustness of these findings. Third, methodologically, as a cross-sectional study design, while it reveals spatial correlation patterns among variables, it cannot rigorously verify causal temporal relationships between factors. Furthermore, the failure to fully account for temporal dynamics of influencing factors may affect the timeliness and explanatory power of the model results. These limitations suggest directions for future research improvements, including adopting multi-scale analytical frameworks combined with longitudinal tracking data to strengthen causal inference capabilities.

## 5 Conclusion

This study systematically reveals the spatial differentiation characteristics of regional life expectancy in China and its underlying formation mechanisms. Empirical analyses demonstrate that the spatial distribution of life expectancy exhibits a distinct east-high, west-low pattern, with significant spatial clustering effects—manifested as two typical spatial patterns: high-high clusters and low-low clusters.

A comparative analysis of spatial regression models confirms that the MGWR model demonstrates superior performance in capturing the spatial heterogeneity of influencing factors, with better fitting accuracy than traditional spatial regression methods. The study identifies years of education as the core determinant of regional life expectancy, with stronger enhancing effects observed in western regions. Similarly, GDP per capita exerts the greatest influence on life expectancy in western China. Conversely, increased household size has an inhibitory effect on life expectancy in certain western areas.

Notably, the negative correlation between the quantity of medical resources and life expectancy—particularly pronounced in southwestern regions—suggests potential inefficiencies in current healthcare resource allocation. More crucially, significant synergistic enhancement effects exist among influencing factors, with the interaction between economic development and education level being particularly prominent. This synergistic effect surpasses the independent impact of any single factor.

The findings not only deepen the understanding of the mechanisms behind regional health disparities but also provide a scientific basis for formulating differentiated regional health promotion strategies.

## Data Availability

The original contributions presented in the study are included in the article/Supplementary material, further inquiries can be directed to the corresponding author.
